# Functional Reorganization of Motor and Limbic Circuits after Exercise Training in a Rat Model of Bilateral Parkinsonism

**DOI:** 10.1371/journal.pone.0080058

**Published:** 2013-11-21

**Authors:** Zhuo Wang, Kalisa G. Myers, Yumei Guo, Marco A. Ocampo, Raina D. Pang, Michael W. Jakowec, Daniel P. Holschneider

**Affiliations:** 1 Department of Psychiatry and Behavioral Sciences, University of Southern California, Los Angeles, California, United States of America; 2 Department of Neurology, University of Southern California, Los Angeles, California, United States of America; 3 Department of Cell and Neurobiology, University of Southern California, Los Angeles, California, United States of America; 4 Department of Biomedical Engineering, University of Southern California, Los Angeles, California, United States of America; Universidade do Estado do Rio de Janeiro, Brazil

## Abstract

Exercise training is widely used for neurorehabilitation of Parkinson’s disease (PD). However, little is known about the functional reorganization of the injured brain after long-term aerobic exercise. We examined the effects of 4 weeks of forced running wheel exercise in a rat model of dopaminergic deafferentation (bilateral, dorsal striatal 6-hydroxydopamine lesions). One week after training, cerebral perfusion was mapped during treadmill walking or at rest using [^14^C]-iodoantipyrine autoradiography. Regional cerebral blood flow-related tissue radioactivity (rCBF) was analyzed in three-dimensionally reconstructed brains by statistical parametric mapping. In non-exercised rats, lesions resulted in persistent motor deficits. Compared to sham-lesioned rats, lesioned rats showed altered functional brain activation during walking, including: 1. hypoactivation of the striatum and motor cortex; 2. hyperactivation of non-lesioned areas in the basal ganglia-thalamocortical circuit; 3. functional recruitment of the red nucleus, superior colliculus and somatosensory cortex; 4. hyperactivation of the ventrolateral thalamus, cerebellar vermis and deep nuclei, suggesting recruitment of the cerebellar-thalamocortical circuit; 5. hyperactivation of limbic areas (amygdala, hippocampus, ventral striatum, septum, raphe, insula). These findings show remarkable similarities to imaging findings reported in PD patients. Exercise progressively improved motor deficits in lesioned rats, while increasing activation in dorsal striatum and rostral secondary motor cortex, attenuating a hyperemia of the zona incerta and eliciting a functional reorganization of regions participating in the cerebellar-thalamocortical circuit. Both lesions and exercise increased activation in mesolimbic areas (amygdala, hippocampus, ventral striatum, laterodorsal tegmental n., ventral pallidum), as well as in related paralimbic regions (septum, raphe, insula). Exercise, but not lesioning, resulted in decreases in rCBF in the medial prefrontal cortex (cingulate, prelimbic, infralimbic). Our results in this PD rat model uniquely highlight the breadth of functional reorganizations in motor and limbic circuits following lesion and long-term, aerobic exercise, and provide a framework for understanding the neural substrates underlying exercise-based neurorehabilitation.

## Introduction

There is now extensive evidence in animals that exercise training (ET) can induce both structural and functional adaptation (“plasticity”) within motor areas, including the motor cortex [1], [2], basal ganglia [3]–[5], cerebellum [6], [7], and red nucleus [8]. Acquired motor behaviors can endure in the absence of continued training, suggesting that motor learning is persistently encoded within the nervous system.

Treadmill training in subjects with Parkinson’s disease (PD), a disorder characterized by dopaminergic cell loss and basal ganglia injury, has been shown in numerous studies to improve motor symptoms [9]–[13]. While we know much about functional brain activation during the execution of acute motor tasks [14]–[17] and motor learning of hand and finger tasks [18] mainly from studies involving healthy subjects, substantially less is known about the functional reorganization of the brain after long-term aerobic exercise, particularly in PD patients. Indeed, most studies on extensive motor training in human subjects have focused on the learning of finger tasks during an fMRI imaging session (reviewed in [19], [20]), with a near absence of studies examining the effects of long-term ET on functional brain changes [21], [22]. An improved understanding of the effects of long-term aerobic training on brain functional activation, in particular in PD patients, would be helpful for directing targeted neurorehabilitative strategies, and in understanding motor compensations in PD.

The current study provides an in-depth, voxel-based, whole brain analysis of functional brain reorganization in a rodent model. Dopaminergic deafferentation is elicited with the neurotoxin 6-hydroxydopamine (6-OHDA), followed by study of the effects on brain function of 4 weeks of forced running wheel exercise. In order to address gait-induced brain circuitry activation, we performed functional brain mapping in awake rats, both during horizontal treadmill walking, as well as at rest. We tested the hypothesis that 4 weeks of exercise training improves motor deficits and normalizes functional deficits in the corticostriatal circuit. Because of the important interaction of the basal ganglia and the limbic system [23], and in light of a robust literature documenting the presence of mesolimbic dopaminergic deafferentation in human PD subjects, we also examined the effects prolonged exercise has on alterations in regions of the mesolimbic circuit [24], [25].

## Materials and Methods

### Ethics Statement

This study was carried out in strict accordance with the recommendations in the Guide for the Care and Use of Laboratory Animals of the National Institutes of Health. The protocol was approved by the Institutional Animal Care and Use Committee of the University of Southern California (#A3518-01). The animal facility at this Institution is accredited by the Association for Assessment and Accreditation of Laboratory Animal Care (AAALAC), International.

### Animals

Adult, male Sprague-Dawley rats (3 months old at the time of stereotaxic surgery) were purchased from Harlan Laboratories (Indianapolis, IN, USA) and housed in pairs on a 12-hr light/12-hr dark cycle with free access to water and rodent chow.

### Overview of the Experimental Protocol

**Figure 1:**
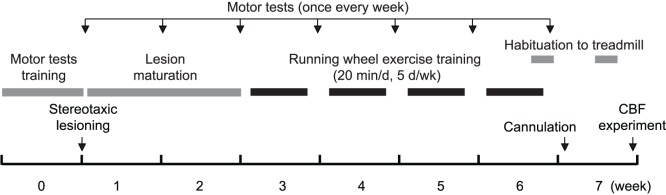
Timeline of experiments.

As shown in Fig. 1, the animals were trained with motor tests, and their baseline motor performance measured prior to the stereotaxic surgery. Motor test performance was measured once a week thereafter. Animals were allowed two weeks of recovery for the lesion to mature. Starting on week 3, rats were exposed to forced exercise training (ET) or sham training (No-ET) for 4 weeks. At the beginning of week 7, animals were intravenously cannulated and allowed to recover for 4 days. Cerebral blood flow (CBF) mapping experiments were performed at the end of week 7 while the animals were either walking on a treadmill or resting. Whole brain sectioning was performed, followed by autoradiography and tyrosine hydroxylase (TH) staining for the quantification of lesion volume. Three different locomotor devices were used: a rotarod (for testing of motor deficits), a running wheel (for ET), and a horizontal treadmill (for CBF imaging during walking). This was to control different levels of device and task familiarity as a confounding factor for motor tests and CBF mapping. Animals were randomized into the following groups: Lesion/Walk/ET (*n* = 11), Lesion/Rest/ET (*n* = 12), Lesion/Walk/No-ET (*n* = 9), and Lesion/Rest/No-ET (*n* = 10). Comparison was made to sham animals without exercise exposure (Sham/Walk/No-ET, *n* = 10); Sham/Rest/No-ET *n* = 9). This allowed us to address the issue of functional normalization in the lesioned, exercised animals. The effect of ET was assessed in lesioned animals. We did not examine sham animals with exercise training because of the difficulty in creating an ‘equivalent’ control exercise intensity between lesioned and sham-lesioned animals. Pilot experiments showed that sham-lesioned rats reached a peak running speed more than twice as high as that of the lesioned rats following the same training protocol. A detailed investigation of the effect of ET on functional brain activation in non-lesioned rats was beyond the scope of the current study. For discussion of behavioral and immunohistochemical data, the above groups were merged into 3 large groups, Lesion/ET (*n* = 23), Lesion/No-ET (*n* = 19), and Sham/No-ET (*n* = 19).

### Animal Model and Stereotaxic Surgical Procedure

The 6-OHDA basal ganglia injury model is a widely accepted model of dopaminergic deafferentation, and while not identical to PD, parallels the human disorder remarkably well [26]. To prevent any noradrenergic effects of the toxin, animals received desipramine (25 mg/kg in 2 mL/kg bodyweight in saline, i.p., Sigma-Aldrich Co., St. Louis, MO, USA) before the start of surgery [27]. They were then placed under isoflurane anesthesia (1.5% in 30% oxygen and 70% nitrous oxide) in a stereotaxic apparatus (David KOPF Instruments, Tujunga, CA, USA) and received injection of 6-OHDA (Sigma-Aldrich Co.) at four injection sites targeting the dorsal striatum bilaterally (AP: +0.6, L: ±2.7, V: −5.1 mm, and AP: −0.4, L: ±3.5, V: −5.5 mm, relative to the bregma). Injection of 10 µg of 6-OHDA dissolved in 2 µL of 1% L-ascorbic acid/saline was made at each site through a 10 µL Hamilton microsyringe (Hamilton Company, Reno, NV, USA) fitted with a 26 gauge, blunted needle, at 0.4 µL/min controlled by a Micro4 microsyringe pump controller (World Precision Instruments, Sarasota, FL, USA). Sham-lesioned rats received 4 injections of an equal volume of vehicle. After injection, the needle was left in place for 5 min before being slowly retracted (1 mm/min).

### Cannulation Surgery

At the beginning of week 7, animals were anesthetized (isoflurane 2% in 30% oxygen and 70% nitrous oxide). The right external jugular vein was cannulated with a 5 French silastic catheter (Dow Corning Corp., Midland, MI, USA), advanced into the superior vena cava. The port at the distal end of the catheter was tunneled subcutaneously and externalized dorsally in the region rostral to the scapula. All animals were allowed to recover for 4 days. Postoperatively, the catheter was flushed daily to ensure patency (0.3 mL of sterile 0.9% saline, followed by 0.1 mL saline containing 40 unit/mL heparin).

### Assessment of Motor Deficits

#### Accelerating rotarod

Rats were familiarized with the rotarod (Columbus Instruments, Columbus, OH, USA) starting 1 week before stereotaxic surgery. For 2 consecutive days, rats were run on the rotarod at 10 rpm (2.29 m/min) for 3 min each day. For the next 3 days, rats were run using an acceleration paradigm (initial speed = 5 rpm = 1.15 m/min, acceleration = 6 rpm/min = 1.38 m/min^2^, 2 trials/day, 30 min. intertrial interval) until they fell or reached the 5 min cutoff time (maximum speed = 35 rpm = 8.02 m/min).

#### Rearing

No prior training was required for this test. The rearing test was always the first motor test of the day. The room was illuminated with lights from an adjacent room through a half opened door. The animal was habituated to the room for 15 min. It was then put in an arena for 5 min. The number of rearings was counted manually from video recordings. A rearing was defined as when the animal lifted both forepaws off the ground. To maintain the level of novelty, arenas of different shapes and wall decorations were used. Olfactory cues were minimized by wiping the arena with 1% ammonia solution between animals.

#### Beam crossing

Rats were familiarized with the paradigm 1 week prior to the surgery. A beam was placed horizontally and fixed with tape between a small platform (6×8 cm) and the edge of a bench, 80 cm apart. The beam was 50 cm from the floor. A Plexiglas black box (L: 30, W: 15, H: 15 cm) was place on the edge of the bench with its opening facing the small platform. The room light was turn off and a lamp illuminated the small platform from 50 cm above. For a trial of beam crossing, the animal was put on the small platform with its head facing the black box. The time for the animal to cross the 80 cm distance was recorded. The animal was left in the box for 20 s at the end of each trial. On the first day of training, the animal was first put into the black box and allowed to explore for 20 s. It was then trained for 3 trials using a 2.5×2.5 cm square beam, followed by 3 trials using a 1.3×1.3 cm square beam, with a 3 min intertrial interval. The animal was guided by the experimenter during the first few trials if necessary. Over the next 5 days of training, as well as in future weekly testing, the animal was tested for 3 trials/session using the 1.3×1.3 cm square beam, followed by 3 trials using a round beam (diameter = 1.3 cm) with a 3 min intertrial interval each day. In a few trials when lesioned rats failed to complete the round beam crossing test by falling off the beam, the time to cross was recorded as 300% of the baseline.

#### Adjusting footstep

For 3 days before baseline testing, rats were habituated to the handling by the experimenter for 3 min/day, including the grip as described below. Baseline data were measured for the next two days. The experimenter held the rat with one hand fixing the hindlimbs with a towel, and the other fixing the forelimb not tested. The hind part of rat was slightly raised above the table surface with the tested paw touching the surface and bearing some bodyweight. The rat was then moved slowly sideways (5 s for 0.9 m), first in the forehand and then in the backhand direction. The number of adjusting footsteps was counted for both forepaws in the backhand and forehand directions of movement. The test was repeated twice each day with a 5 min intertrial interval.

#### Spontaneous locomotor activity

Shortly before 6 pm on the day of testing, animals were put individually in a new cage with new bedding. Activity counts of each rats were recorded in the horizontal plane for 12-hours (6 p.m.–6 a.m.) by an infrared beam break system (Opto-M3, Columbus Instruments, Columbus, OH) mounted around their cages. Activity was recorded prior to lesioning, as well as weekly thereafter. Locomotor activity data for 1 rat in the Lesion/No-ET, 1 in the Sham/No-ET, and 2 in the Lesion/ET group were not collected due to equipment failure.

#### Forced exercise training

**Figure 2:**
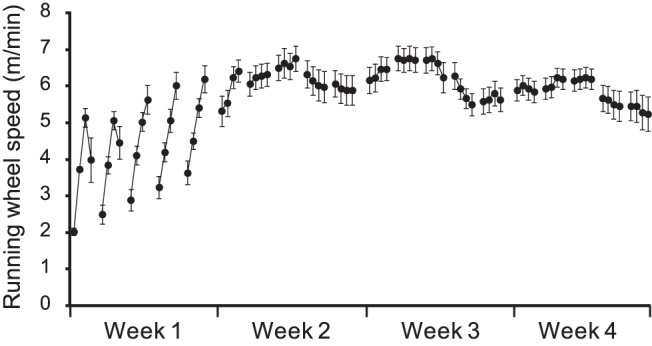
Training speeds during 4 weeks of forced running wheel exercise. Lesioned rats received four 5-min exercise sessions/day (2 min. intersession interval) and 5 consecutive days/week beginning 2 weeks after bilateral striatal lesioning.

Starting in week 3, animals assigned to ET groups were trained in a running wheel for 20 min/day, 5 consecutive days/week for 4 weeks (Fig. 1). No-ET animals were handled and left in a stationary running wheel for 30 min/day. The running wheels (35.6 cm diameter, Lafayette Instrument, Lafayette, IN, USA,) were modified by adding an inner plastic ‘floor’ to cover the metal rungs. Pilot experiments showed this modification made it easier for lesioned rats to learn. On the day of training, the animal was placed in the running wheel for 5 min, followed by four 5-min running sessions, with 2-min inter-session intervals. During the 1^st^ week of ET, animals were habituated to the wheels and trained with a starting speed of 2 m/min. Speed was ramped up with either 1 or 2 m/min increments from session to session within a day and the starting speed was progressively increased from day to day depending on how fast the animal learned. Starting with the 2^nd^ week of ET, we applied an adaptively challenging paradigm for ET training (see Fig. 2). The purpose was to train the animal at the highest speed possible without causing significant stress. Defecation and urination were monitored as signs of acute stress responses. Failure was defined as the animal falling behind the speed of the turning wheel, allowing its body to reach a near vertical position at the back of the wheel before getting tossed over (with both hindpaws airborne). The following rules were applied in adjusting the speed. If the animal had no more than 1 failure and showed no sign of stress over 2 consecutive sessions, the speed was increased by 1 m/min. If the animal had more than 6 failures in a single session, the session was ended and the speed was decreased by 1 m/min for the next session. Following vascular cannulation and two days prior to the CBF experiments, rats were exercised for two 5-min sessions at half of the training speed achieved at the end of week 6.

#### Brain mapping

All animals were habituated to a horizontal treadmill for 4 days: 2 days at the end of week 6 and 2 days prior to cerebral perfusion experiments. They were individually placed on the stationary treadmill (L 50, W 7, H 30 cm) for 10 min followed by 3 min of walking at 8 m/min. On the day of the perfusion experiment, the animal was allowed to rest in the treadmill for 10 min. A piece of silastic tubing was filled with radiotracer [^14^C]-iodoantipyrine (125 µCi/kg in 300 µL of 0.9% saline, American Radiolabelled Chemicals, St. Louis, MO, USA). The radiotracer-filled tubing was then connected to the animal’s cannula on one end, and to a syringe filled with euthanasia agent (pentobarbital 50 mg/mL, 3 M potassium chloride) on the other. The animal was allowed to rest for another 5 min. For animals assigned to the ‘walking’ condition for CBF, the treadmill was then turned on and set at 8 m/min, while it remained off for animals assigned to the ‘rest’ condition for CBF. After 2 min, radiotracer was infused at 2.25 mL/min by a motorized pump, followed immediately by 0.7 mL of the euthanasia solution, which resulted in cardiac arrest within ∼10 s, a precipitous fall of arterial blood pressure, termination of brain perfusion, and death. This approach uniquely allowed a 3-dimensional assessment of functional activation in the awake, unrestrained animal, with a temporal resolution of ∼5–10 sec. and an in-plane spatial resolution of 100 µm^2^. [28], [29] Time of day and duration of testing, persons performing the testing, lighting, ambient sound levels, room temperature were kept constant between sessions. Olfactory cues were minimized by wiping the treadmill with a 1% ammonia solution.

#### Autoradiography

Brains were removed, flash frozen at −55°C in dry ice/methylbutane and serially sectioned for autoradiography (57 coronal 20-µm slices, 300-µm interslice distance beginning at 4.7 mm anterior to bregma). Autoradiographic images of brain slices along with 12 [^14^C] standards (Amersham Biosci.) were digitized on an 8-bit gray scale using our prior methods [30], [31]. CBF related tissue radioactivity was measured by the classic [^14^C]-iodoantipyrine method [32]–[34]. In this method, there is a strict linear proportionality between tissue radioactivity and CBF when the data is captured within a brief interval (∼10 seconds) after the tracer injection. [35], [36].

#### Image analysis

A 3-dimensional reconstruction of each animal’s brain was conducted using 57 serial coronal sections (starting at ∼ bregma +4.5 mm) with a voxel size of 40 µm×300 µm×40 µm [31]. Adjacent sections were aligned manually in Photoshop (version 9.0, Adobe Systems Inc., San Jose, CA, USA) and using TurboReg, an automated pixel-based registration algorithm implemented in ImageJ (version 1.35, http://rsbweb.nih.gov/ij/). This algorithm registered each section sequentially to the previous section using a nonwarping geometric model that included rotations and translations (rigid-body transformation) and nearest-neighbor interpolation. One “artifact free” brain was selected as reference. All brains were spatially normalized to the reference brain by statistical parametric mapping (SPM, version 5, Wellcome Centre for Neuroimaging, University College London, London, UK). Spatial normalization consisted of applying a 12-parameter affine transformation followed by a nonlinear spatial normalization using 3-D discrete cosine transforms. All normalized brains were then averaged to create a final rat brain template. Each original 3D-reconstructed brain was then spatially normalized to the template. Normalized brains were smoothed with a Gaussian kernel (FWHM = 3×voxel dimension). Voxels for each brain failing to reach a specified threshold in optical density (70% of the mean voxel value) were masked out to eliminate the background and ventricular spaces without masking gray or white matter. To account for any global differences in the absolute amount of radiotracer delivered to the brain, adjustments were made by the SPM software in each animal by scaling the voxel intensities so that the mean intensity for each brain was the same (proportional scaling). Prior work has demonstrated that 6-OHDA striatal lesions in rats result in no significant difference in absolute cerebral blood flow (mL/g/min) [37], [38]. A nonbiased, voxel-by-voxel Student’s t-test analysis was performed to evaluate the effects of treadmill walking versus rest, lesion versus sham-lesion, and exercise versus no-exercise. Threshold for significance was set at *P*<0.05 at the voxel level and an extent threshold of 100 contiguous voxels. This combination reflected a balanced approach to control both type I and type II errors. The minimum cluster criterion was applied to avoid basing our results on significance at a single or small number of suprathreshold voxels. Brain regions were identified according to a rat brain atlas [39]. In addition, we ran factorial analyses to identify rCBF differences reflecting the Lesion×Walk interaction among the 4 groups of nonexercise rats (Lesion/Walk/No-ET, Lesion/Rest/No-ET, Sham/Walk/No-ET, Sham/Rest/No-ET), and the Exercise×Walk interaction among the 4 groups of lesioned rats (Lesion/Walk/ET, Lesion/Rest/ET, Lesion/Walk/No-ET, Lesion/Rest/No-ET). Threshold for significance was set at *P*<0.05 at the voxel level and an extent threshold of 100 contiguous voxels. Data interpretation was focused on gray matter.

### Tyrosine Hydroxylase Staining of Dopaminergic Neurons

Sections were fixed for 10 min at RT with 2% PFA, rinsed with PBS, and quenched with 0.3% H_2_O_2_+0.3% NGS in PBS for 30 min at RT. After rinsing in PBS, slides were blocked with 4% NGS/x1 PBS and incubated overnight at 4°C in primary antibody solution (1∶2000 anti-tyrosine hydroxylase, clone LNC1, Millipore, Billerica, MA in 2% NGS/x1 PBS). Sections were washed the following day in PBS and incubated in secondary antibody solution (biotinylated anti-mouse IgG, Vectastain Elite ABC kit) 1∶2500 in 2% NGS/x1 PBS, 30 min at RT. Staining was developed with DAB (10 mg DAB+10 µl 30% H_2_0_2_ in 10 mL PBS), until optimal contrast on sections was achieved. Sections were then dehydrated, mounted, and dried overnight.

Volume of the striatum, substantia nigra compacta, substantia nigra reticulata, and cerebral hemisphere were defined bilaterally in the digitized, thresholded images of each rat by manual tracing using ImagePro Plus 4.0 (Media Cybernetics, Rockville, MD)(striatum: 6 coronal sections anterior and 3 sections posterior to bregma, ∼AP+1.56 mm −1.14 mm; substantia nigra: ∼5 coronal sections, AP-5.20 mm −6.40 mm; 300 µm interslice distance). Lesion of the striatum were evaluated both as lesion volume as a percentage of ipsilateral striatal volume or as reduction in the optical density (OD) of the striatum. OD values were normalized in each animal by values obtained in the corpus callosum using the formula 1000 * log_10_(OD_cc_/OD_striatum_). Lesions of the substantia nigra compacta or reticulata were similarly evaluated as changes in normalized optical density.

### Statistical Analysis

Bodyweight, TH staining, and motor tests data were normalized to each animal’s baseline value and presented as percentages (mean ± SEM). To test whether 4 weeks of ET induced changes in motor function and in TH staining, the two-tailed Student’s *t* test was used to compare week 6 data (postmortem data for TH staining) for Lesion/ET and Lesion/No-ET rats. Effects of ET were also analyzed over the 4-week ET period using a repeated measure two-way ANOVA, followed by Sidak *post hoc* tests, with ‘ET’ as the between-subjects factor and ‘Time’ the within-subjects factor. The effects of lesion were tested using a repeated measure two-way ANOVA on data over the 6 week period, with ‘Lesion’ as the between-subjects factor and ‘Time’ the within-subjects factor. To test whether there were differences in bodyweight on the day of CBF experiments, one-way ANOVA test was performed followed by Sidak *post hoc* tests. The analysis was performed in Prism (version 6, GraphPad Software, La Jolla, CA, USA). *P*<0.05 was considered statistically significant.

## Results

### Effects of Lesions and Exercise on Bodyweight, Tyrosine Hydroxylase Staining, and Motor Function

Following the intrastriatal injection of 6-OHDA, rats lost an average of 10% bodyweight 4 days after the surgery (90±1%, *n* = 42), consistent with prior reports of lesion-associated dysphagia and weight loss [40]. In comparison, sham rats showed maximum weight loss of 3% 2 days after the surgery (97±1%, *n* = 19). Thereafter, all animals gradually gained bodyweight, including during the weeks of ET. On the day of CBF experiments, bodyweights were (116±2) % for the Lesion/No-ET rats, (114±1) % for the Lesion/ET rats, and (119±1) % for the Sham/No-ET rats. There were significantly differences in bodyweight (*F*(2,58) = 4.59, *P* = 0.01, one-way ANOVA). Post hoc test showed significant difference only between the Lesion/ET and the Sham/No-ET rats (*P*<0.05). The relatively small differences in bodyweight among groups were not expected to substantively affect the rest of the results.

**Figure 3:**
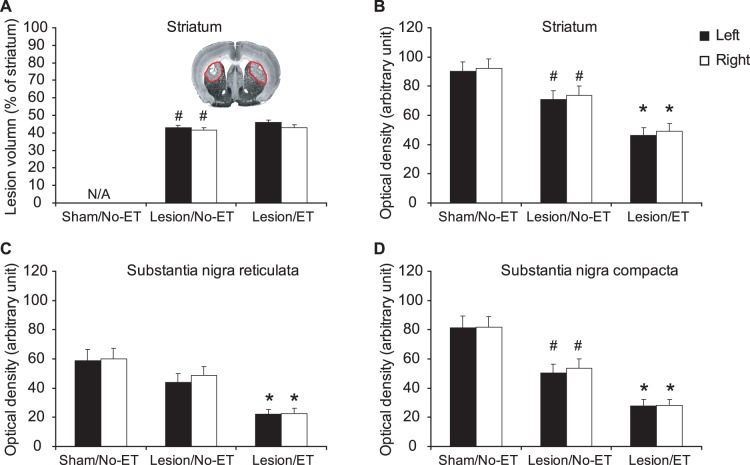
6-OHDA lesions. Shown are the effect of bilateral striatal lesions on (A) lesion volume (% of striatal volume), (B) tyrosine hydroxylase (TH) staining of the striatum, (C) TH staining of the substantia nigra reticulata (SNR), and (D) TH staining of the substantia nigra compacta (SNC). #: significant difference between sham rats without exercise training (Sham/No-ET) and lesioned rats without ET (Lesion/No-ET), *P*<0.05, Student’s *t* test. *: significant difference between Lesion/No-ET and Lesion/ET, *P*<0.01, Student’s *t* test. The inset in (A) shows a representative TH stained coronal section showing bilateral depletion of TH (outlined in red).

Postmortem TH staining showed robust, bilateral striatal lesion measured both as significantly non-zero lesion volume (Fig. 3A) and a significant decrease in striatal TH optical density 7 weeks following lesioning (Fig. 3B). While ET had no significant effect on lesion volume in lesioned rats (Fig. 3A), a significant decrease in striatal TH optical density was noted in Lesion/ET compared to Lesion/No-ET animals (Fig. 3B). In the substantia nigra, lesioning resulted in significant decreases in TH optical density bilaterally in the compacta (SNC) (Fig. 3D), but not in the reticulata (SNR) (Fig. 3C). In lesioned rats, ET resulted in significant decreases in TH optical density in both SNC and SNR (Fig. 3C,D).

**Figure 4:**
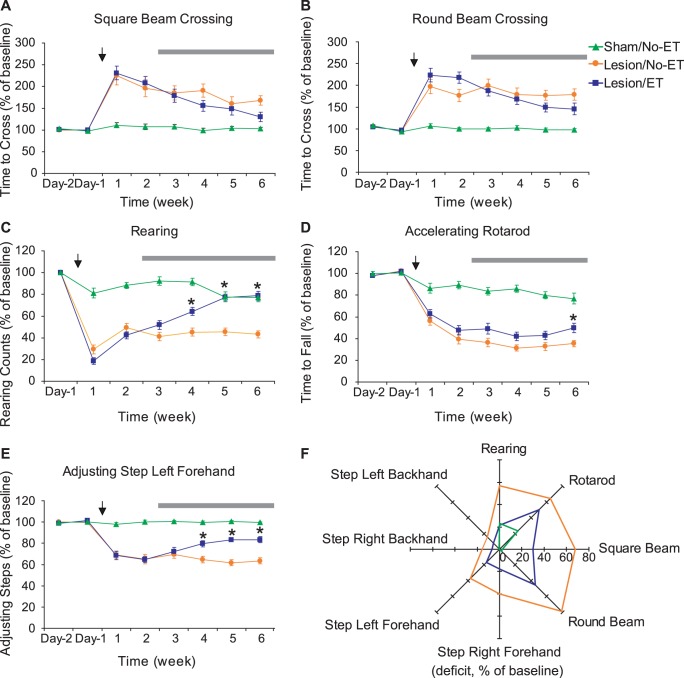
Striatal lesioning-induced motor deficits and exercise training (ET)-induced recovery in motor function. (A–E) Time course of motor tests results. Main effect of lesion was analyzed with repeated measure two-way ANOVA comparing Sham/No-ET (*n* = 19) and Lesion/No-ET (*n* = 19) groups over the 6 weeks period after lesioning (time of lesioning was denoted with the arrows). Lesion-induced motor deficits were significant in all motor tests, and *post hoc* tests showed significant lesion-induced deficits at all time points. Main effect of ET was analyzed with repeated measure two-way ANOVA comparing Lesion/No-ET and Lesion/ET (*n* = 23) groups over the 4 weeks ET period (denoted by the horizontal bars). ET-induced improvements were significant for the rearing, accelerating rotarod, and adjusting step tests, with nonsignificant trends shown in the beam crossing tests. *: signifcant effect of ET by *post hoc* tests (*P*<0.05). Only data for the left forehand adjusting step test were shown, which were representative of all four adjusting step tests. (F) The radar graph depicts the deficit (as a % of prelesion baseline) for each motor test at the 6 week time point. Four-week ET induced statistically significant improvement in all motor tests (*P*<0.05, Student’s t-test) except the round beam test (*P* = 0.09).

Intrastriatal 6-OHDA lesion caused deficits in all motor tests (Fig. 4). Deficits in the two beam crossing tests and the rearing test peaked 1 week following the surgery and improved slightly to a plateau level 2 weeks after the surgery (Fig. 4A, B, C. Main lesion effects: square beam, *F*(1,36) = 34.28, *P*<0.0001; round beam, *F*(1,36) = 53.76, *P*<0.0001; rearing, *F*(1,36) = 94.26, *P*<0.0001, repeated measure two-way ANOVA. *Post hoc* tests showed significant lesion-induced deficits at all time points, as for all motor tests.) Deficits in the adjusting step tests reached a plateau 1 week after the surgery (Fig. 4E showed data for the left forehand, which were representative of all four adjusting step tests. *F*(1,36) = 141.9, *P*<0.0001), whereas deficits in the accelerating rotarod test peaked ∼2 weeks after the surgery and remained relatively stable for the rest of the study (Fig. 4D. *F*(1,36) = 127.2, *P*<0.0001). Lesion did not cause changes in overnight homecage locomotor activity counts (*F*(1,34) = 0.28, *P* = 0.6).

Compared to lesioned rats without exercise, 4 weeks of forced exercise training ameliorated lesion-induced motor deficits in all motor tests (Fig. 4F) except the round beam test (*P* = 0.09, Student’s *t* test). When all data over the 4-week ET period were analyzed with two-way ANOVA, significant main ET effects were found in rearing (*F*(1, 40) = 37.96, *P*<0.0001), accelerating rotarod (*F*(1,40) = 5.95, *P* = 0.02), and adjusting step left forehand (*F*(1,40) = 15.55, *P* = 0.0003), but only trend of effects in square beam (*F*(1,40) = 1.83, *P* = 0.19) and round beam crossing tests (*F*(1,40) = 1.92, *P* = 0.17). Overnight locomotor activity was not significantly affected by ET (*F*(1,37) = 0.24, *P* = 0.6).

### Changes in rCBF during Walking and Effects of 6-OHDA Lesions (Fig. 5, Tables 1, 2, “Walk vs. Rest, Lesion/No-ET” and “Walk vs. Rest, Sham/No-ET”)

Both lesioned and sham-lesioned rats easily performed the locomotor challenge, walking at a slow rate on the horizontal treadmill without footslips. In both sham and lesioned animals, walking compared to rest resulted in increased rCBF in primary and secondary motor cortex and the cerebellar vermis, with deactivation noted in the substantia nigra, globus pallidus (external, internal), subthalamic n., thalamus (ventrolateral, ventromedial, central medial), trigeminal n. (motor, sensory) and associated motor regions (red n., superior colliculus, pedunculopontine tegmental n.). Also noted was a significant increase in rCBF of the primary somatosensory (dysgranular), parietal association and visual cortices (primary, secondary), and a significant decrease of rCBF of primary somatosensory cortex (jaw, upper lip), as well as anterior secondary somatosensory cortex. Differences in functional brain response were noted at the level of the striatum. During walking, sham animals increased rCBF in the anterior medial striatum (rostral to the fusion of the corpus callosum) and the dorsal/dorsolateral striatum (extending from the fusion of the corpus callosum to the start of the hippocampus). Lesioned rats, on the other hand, showed a decreased rCBF broadly throughout the striatum. Activation of the zona incerta was seen only in lesioned rats but not in sham animals.

In limbic and paralimbic brain regions of both lesioned and sham-lesioned rats, the locomotor challenge resulted in increased rCBF in the hippocampus (dorsal, ventral), ventral subiculum, septum (medial, lateral), medial prefrontal cortex (cingulate, prelimbic, infralimbic), dorsal peduncular cortex, anterior retrosplenial cortex (posterior cingulate), orbital cortex (lateral, ventral) and entorhinal cortex, while decreased rCBF was noted within the amygdala (central n., basolateral n., lateral n.), ventral striatum (anterior to the hippocampus), periaqueductal gray, raphe and insula.

### Effects of Bilateral Striatal Lesions on rCBF (Fig. 5; Tables 1–3, “Lesion vs. Sham, Walk/No-ET” and “Lesion vs. Sham, Rest/No-ET”)

Lesioned compared to sham-lesioned rats demonstrated significant differences in rCBF that were similar in distribution when imaged either at rest or during the treadmill walking. This included a significantly decreased rCBF response in the dorsal striatum and secondary motor cortex (rostral, caudal), as well as compensatory increases in rCBF in the globus pallidus (external, internal), thalamus (ventromedial, ventroposterior lateral/medial, central medial), associated motor regions (red n., superior colliculus) and zona incerta. Different was the response in the anterior striatum, primary motor cortex, and cerebellar vermis, where lesioned compared to sham animals showed a decreased rCBF during walking, while an increased rCBF or no change was noted at rest. Increased rCBF was noted in the anterior-medial striatum only at rest and in the deep cerebellar nuclei (medial, lateral, interposed) during treadmill walking. Factorial analysis confirmed the main effect of Lesion in the striatum (anterior, dorsal), secondary motor cortex (rostral, caudal), globus pallidus (internal, external), thalamus (central medial, ventromedial, ventroposterior lateral/medial nuclei), red nucleus, superior colliculus, deep cerebellar nuclei (medial, lateral, interposed) and vermis. The Lesion×Walk interaction was significant in the primary and secondary motor cortex (rostral), anterior and dorsal striatum, superior colliculus, thalamus (ventrolateral, ventral anterior nuclei), and cerebellar vermis (*F*_1,34_ = 4.13, *P*<0.05).

Lesioned compared to sham-lesioned rats also showed a functional reorganization of broad cortical regions, with a significant increased rCBF response, both at rest and during walking, in primary somatosensory cortex (jaw region, upper lip), secondary somatosensory cortex and auditory cortex. Significantly decreased responses were noted at rest and during walking in ectorhinal, secondary visual and parietal association cortex. Factorial analysis confirmed the main effect of Lesion in the primary somatosensory cortex (forelimb, jaw region, upper lip), anterior secondary somatosensory and ectorhinal cortex. The Lesion×Walk interaction was significant in primary somatosensory cortex of the forelimb and trunk, secondary somatosensory cortex, ectorhinal cortex, and primary visual cortex (*F*_1,34_ = 4.13, *P*<0.05).

In limbic and paralimbic brain regions, lesions resulted in increased rCBF in several regions, including the amygdala (central n., basolateral n., amygdaloid-piriform and amygdaloid-hippocampal transition area), dorsal peduncular cortex, nucleus accumbens, raphe n., septum (medial, lateral), tegmental nucleus, ventral hippocampus, ventral striatum, insula, piriform and entorhinal cortex, both at rest and during the locomotor challenge. rCBF was significantly decreased in lesioned compared to sham animals in the dorsal hippocampus and retrosplenial cortex at rest and during walking. There was no significant effect of lesioning on activation of the medial prefrontal cortex (cingulate, prelimbic, infralimbic) or orbital cortex. Factorial analysis confirmed the main effect of Lesion in the amygdala (basolateral n., central n., amygdaloid-piriform and amygdaloid-hippocampal transition areas), dorsal endopiriform nucleus, dorsal and ventral hippocampus, ventral striatum, nucleus accumbens, raphe, septum (medial, lateral), tegmental nucleus, insula, entorhinal, piriform and retrosplenial cortex. The Lesion×Walk interaction was significant in the amygdala (central n., basolateral n., lateral n., medial, cortical amygdala), dorsal endopiriform nucleus, ventromedial hypothalamus, ventral striatum, nucleus accumbens, dorsal and ventral hippocampus, and post- and ventral subiculum, insula, piriform and retrosplenial cortex (*F*_1,34_ = 4.13, *P*<0.05).

### Effects of Exercise Training on rCBF of Lesioned Rats (“Exercise vs. No Exercise, Lesion/Rest” and “Exercise vs. No Exercise, Lesion/Walk”; Figs. 6–7, Tables 1-3-3)

Exercise compared to no-exercise in lesioned animals resulted in significantly increased rCBF during walking in regions of the motor circuit, including the globus pallidus (external, internal), dorsal striatum, the thalamus (ventral anterior n., ventral lateral n., ventromedial n., ventroposterior lateral/medial n., reticular n.), the pedunculopontine tegmental n., and secondary motor cortex (rostral). In contrast, when examined at rest, rCBF in these regions was either significantly decreased or showed no change. In addition, exercised compared to nonexercised lesion rats demonstrated significantly decreased rCBF during the locomotor challenge in the vermis, in the cerebellar deep nuclei (medial, lateral, interposed) and zona incerta. Significantly increased rCBF at rest was noted in the anterior-medial striatum and zona incerta. In the walk-vs.-rest comparison, the exercise-related significant increases in rCBF of lesioned animals were particularly apparent in the dorsal striatum and secondary motor cortex (rostral). In addition, a hyperemia of the zona incerta noted in the lesioned, nonexercised animal was not apparent in those animals that had undergone ET. Factorial analysis confirmed the main effect of Exercise in rostral secondary motor cortex, dorsal striatum, internal globus pallidus, pedunculopontine tegmental nucleus, thalamus (ventrolateral n., ventral anterior n.), and superior colliculus. The Exercise×Walk interaction was significant at these sites, as well as in the external globus pallidus, red nucleus, anterior-medial striatum, ventroposterior lateral/medial thalamus, zona incerta, and primary motor cortex (*F*_1,38_ = 4.10, *P*<0.05). The main effect of Exercise was significant in the cerebellar vermis, while the Exercise×Walk interaction was significant in the deep cerebellar nuclei.

Exercise training resulted in different patterns of rCBF in limbic/paralimbic regions in the lesioned animal, when examined either during walking or at rest. Thus, for instance, during walking, previous ET resulted in increased rCBF in the amygdala (central n., basolateral n., lateral n.), dorsal hippocampus, postsubiculum, ventral striatum, and insula. However, when examined at rest, most of these regions showed no change or a significant decrease in rCBF. By comparison, a history of ET resulted at rest in significantly increased rCBF in the cortical amygdala, and amygdaloid-piriform/amygdaloid-hippocampal transition area, the dorsal endopiriform nucleus, ventral hippocampus, periaqueductal gray, lateral septum, ventral subiculum, piriform and retrosplenial cortex. Whereas, these regions showed no change or a significant decrease in rCBF when examined during the locomotor challenge. Exercised compared to non-exercised animals showed a significant decrease in rCBF in the medial prefrontal cortex (cingulate, prelimbic, infralimbic), both at rest and during the locomotor challenge. Factorial analysis confirmed the main effect of Exercise in the basolateral and lateral amygdala, dorsal hippocampus, subiculum (post-, ventral), ventral striatum, nucleus accumbens, medial septum, ventral pallidum, tegmental nucleus, piriform, retrosplenial and entorhinal cortex and medial prefrontal cortex (cingulate, prelimbic, infralimbic). The Exercise×Walk interaction was significant in areas including the amygdala (central, basolateral, medial, amygdaloid-piriform and amygdaloid-hippocampal transition area), dorsal endopiriform nucleus, dorsal and ventral hippocampus, lateral periaqueductal gray, lateral septum, subiculum (post-, ventral), insula, orbital, piriform, retrosplenial and entorhinal cortex (*F*_1,38_ = 4.10, *P*<0.05).

## Discussion

We applied functional brain mapping in awake, freely-moving rats to study the effects of bilateral dorsal striatal lesions and ET on the functional activation of brain motor and limbic circuits. Lesioning led to substantial motor deficits and decreased functional activation of the striatum, motor cortex (including rostral motor cortex). At the same time there was increased engagement of nonlesioned motor regions in the affected basal ganglia circuits, a functional recruitment of associated motor regions and regions within the cerebellar-thalamocortical and mesolimbic circuits, as well as a remapping of cortical sensorimotor activity. Exercise training in lesioned rats induced significant recovery of motor function and elicited a functional reorganization in the basal ganglia-thalamocortical circuit, the rostral secondary motor cortex, the zona incerta, and in regions participating in cerebellar-thalamocortical and mesolimbic pathways. These findings provide new insights into the brain mechanisms underlying the neurorehabilitive effect of long-term, aerobic exercise.

### Lesion Volume and Tyrosine Hydroxylase Staining – Effects of Lesions and Exercise

Based on the immunohistochemical and behavioral findings, lesions in our study fell within the scope of so-called ‘moderate and compensated’ lesions [41]. Tyrosine hydroxylase staining showed robust, bilaterally symmetric lesions 7 weeks following lesioning, with, ∼40% of striatal volume affected and a ∼30% loss in TH striatal optical density. Loss of TH staining was noted also bilaterally in the substantia nigra compacta (∼38% loss in optical density) consistent with retrograde cell death from the injured fibers terminating in the dorsolateral striatum, with TH optical density not significantly decreased in the substantia nigra reticulata [42], [43].

While ET had no significant effect on lesion volume, a significant decrease was noted in exercised compared to nonexercised, lesioned animals in TH optical density at the level of both the striatum (∼42% loss) and substantia nigra (∼45% loss, compacta and reticulata). Previous work has reported exercise-related decreases [44], increases [45]–[47], as well as no change [48]–[50] in TH, with the possibility suggested in the literature that differences may be attributable to the time of harvesting tissue following the final exercise exposure [46] or stress effects [49], [51].

### Motor Testing – Effects of Lesions and Exercise

Neither lesioning, nor ET resulted in any significant change in overnight homecage locomotor activity, confirming the lack of gross motor impairments in our animals. However, during motor testing lesioned compared to sham-lesioned rats showed significant motor deficits at each weekly interval throughout the 6 week follow-up period. Figure 4 shows lesioned rats to have a significantly lengthened time to cross the round and square beams, decreased rearing activity, decreased time to remain on the accelerating rotarod, as well as decreased adjusting steps on the forehand challenge. Exercise in lesioned rats resulted in progressively improved motor deficits, which were most significant during rearing, and during the adjusting steps forehand task, especially during the final two weeks of follow-up. Results at the 6-week time point are succinctly summarized in the radar graph of figure 4, which reveals the consistent effects of lesioning and exercise on all motor task, except the round beam test (P = 0.09).

### Effects of Walking (“Walk vs. Rest”) on Functional Activation of the Motorsensory System

The locomotor challenge elicited a functional activation of the motor circuit that was largely symmetric across the hemispheres, and similar in pattern in both sham and lesioned animals. Results in sham rats closely paralleled our prior work, which reported activation of primary and secondary motor cortex, dorsal striatum and the cerebellar vermis during rotarod walking, as well as deactivation in the thalamus (ventrolateral n., ventromedial n., central medial), globus pallidus (external, internal), red nucleus, substantia nigra, subthalamic nucleus, and superior colliculus [31], [52]. In the current study, lesioned rats showed decreases in rCBF that were broader and larger in magnitude than those noted in sham animals (e.g. primary and secondary somatosensory cortex), while increases in rCBF were less prominent, with primary and secondary motor cortical activation more restricted. Clear differences in functional brain responses during walking were noted at the level of the striatum, where lesioned animals decreased rCBF in the anterior/anterior-medial and dorsal striatum, while sham-lesioned rats showed increased rCBF in these regions. In addition, lesioned but not sham-lesioned rats showed a prominent bilateral activation of the zona incerta (ZI), a band of gray matter lying dorsal to the subthalamic nucleus, and which is a target for therapeutic deep brain stimulation in PD patients [53], [54]. Interesting was a distinct bilateral activation, independent of lesioning, in primary somatosensory dysgranular cortex. This region has been proposed to be involved in regulating somatic and motor information related to the movements of limbs around joints and the stretch of muscles and the integration of these at the level of the striatum, thalamus and zona incerta and midbrain (red n., superior colliculus) [55]. This is the first report of functional activation of this region during walking behavior, and was facilitated by our imaging in the awake animal and whole brain, voxel-based data analysis.

### Effects of Striatal Lesions (“Lesion vs. Sham) on Functional Activation of the Motorsensory System

**Figure 5:**
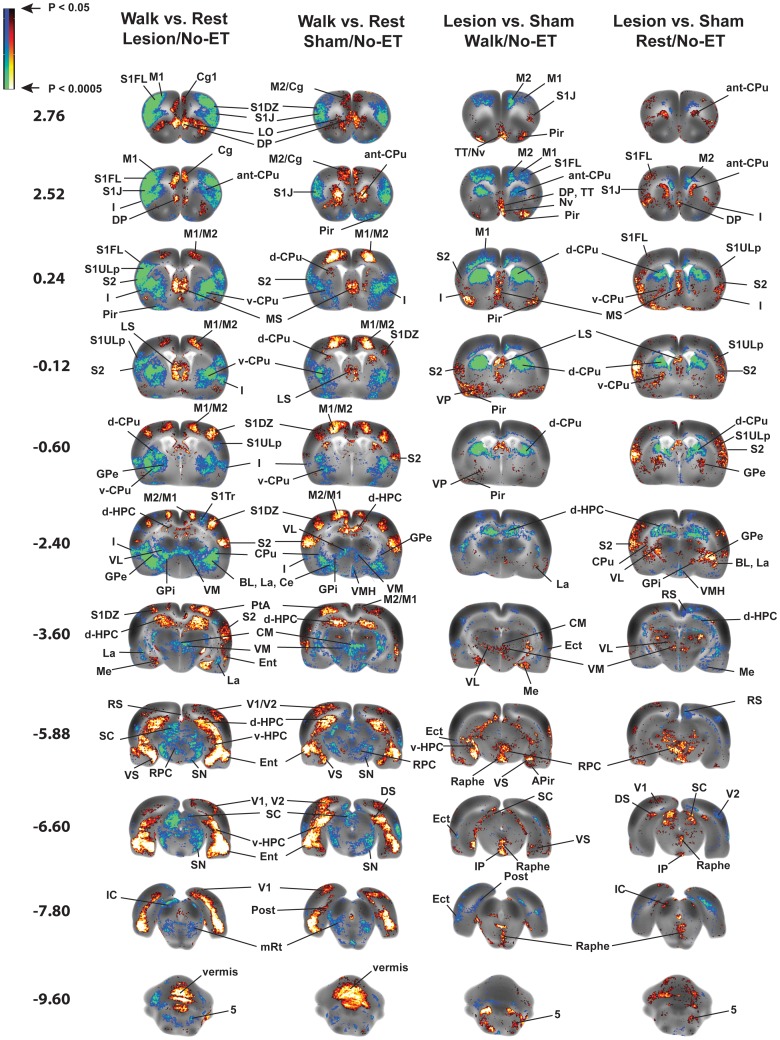
Functional brain activation in rats with bilateral striatal lesions and sham-lesioned rats. Shown are statistically significant differences in activation during acute treadmill walking (Lesion/Walk/No-ET, *n* = 9, Sham/Walk/No-ET, *n* = 10) or at rest (Lesion/Rest/No-ET, *n* = 10, Sham/Rest/No-ET, *n* = 9). Comparison highlights lesion effects (Lesion vs. Sham) or the effect of walking (Walk vs. Rest). No exercise training (ET) was given. Depicted is a selection of representative coronal slices (anterior–posterior coordinates relative to bregma). Colored overlays show statistically significant positive (red) and negative (blue) differences. Abbreviations are those from the Paxinos and Watson rat atlas [39]: 5 (trigeminal n., motor, sensory), aca (anterior commissures), BL (basolateral amygdalar n.), Ce (central amygdalar n.), Cg (cingulate cortex), CM (central medial thalamic n.), CPu (striatum: anterior, ant-CPu; dorsal, d-CPu; ventral, v-CPu), d-HPC (dorsal hippocampus), DP (dorsal peduncular cortex), DS (dorsal subiculum), Ect (ectorhinal cortex), Ent (entorhinal cortex), GPe (external globus pallidus), GPi (internal globus pallidus/entopeduncular n.), I (insular cortex), IC (inferior colliculus), IL (infralimbic cortex), IP (interpeduncular n.), La (lateral amygdalar n.), LO (lateral orbital cortex), LP (lateral posterior thalamic n.), LS (lateral septum), M1, M2 (primary, secondary motor cortex), Me (medial amygdalar n.), MS (medial septum), mRT (mesencephalic reticular formation), Nv (navicular n.), PaS (parasubiculum), PH (posterior hypothalamus), Pir (piriform cortex), Pn (pons), PrL (prelimbic cortex), PtA (parietal association cortex), RPC (red n.), RS (retrosplenial cortex), S1DZ, S1FL, S1J, S1Tr, S1ULp, (primary somatosensory cortex: dysgranular, forelimb, jaw, trunk, upper lip), S2 (secondary somatosensory cortex), SC (superior colliculus), SN (substantia nigra), STN (subthalamic n.), TT (tenia tecta), vermis (2^nd^, 3^rd^ cerebellar simple lobule), V1, V2 (primary, secondary visual cortex), v-HPC (ventral hippocampus), VL (ventral lateral thalamic n.), VM (ventromedial thalamic n.), VMH (ventromedial hypothalamus), VPL/VPM (ventral posterolateral, ventral posteromedial thalamic nuclei), VP (ventral pallidum), VS (ventral subiculum), ZI (zona inserta).

**Figure 7:**
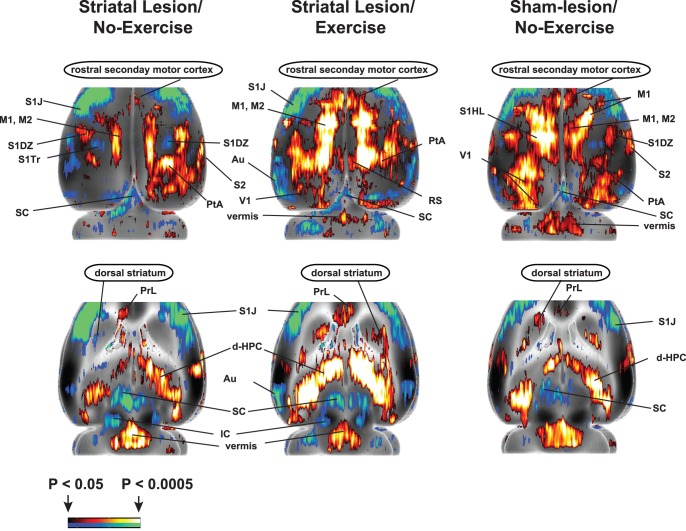
Functional activation of dorsal striatum (d-CPu) and rostral secondary motor cortex (rostral M2). Activation is attenuated after lesioning and is augmented by exercise training (ET). Row 1 depicts a top-down view of the cerebral cortex, while row 2 shows activation at a transverse slice at the level of the dorsal striatum (AP +3.7 mm to AP −10.6 mm). Significant changes are shown for the walk-vs.-rest comparison. Colored overlays show statistically significant positive (red) and negative (blue) differences (voxel level, *P*<0.05). Abbreviations are those noted in the legend of Fig. 5.

The lesioned compared to the sham-lesioned brain showed a number of functional changes, as previously reported in the unilateral striatal lesion model [52]. Functional reorganization included (1) a loss of functional activation at the dorsal striatal lesion site (rest, walking), anterior medial striatum (walking), ventrolateral thalamus (walking), as well as motor cortex (primary, secondary). In particular, lesioned rats compared to sham-lesioned rats showed less activation during both walking and rest in the most rostral motor cortex (Fig. 7), a region with projections to the dorsolateral striatum [56]. Rostral motor cortex in the rodent has been proposed to be the equivalent of the supplementary motor area (SMA) of primates [57]–[59], and in Parkinson’s patients is known to be consistently hypoactive during motor tasks [16]. (2) an increased engagement of nonlesioned neurons in the affected motor circuits, where a relative hyperemia was seen in the internal globus pallidus (entopeduncular n.) (rest, walking) and substantia nigra (rest) – both basal ganglia output nuclei. In addition, hyperemia was noted at rest and during walking in motor-sensory nuclei of the thalamus (ventromedial, ventroposterior lateral/medial, central medial) (Fig. 5). The generalized hyperactivity in regions of the motor circuit was largely in agreement with earlier studies in the 6-OHDA rat using cytochrome oxidase [42], [60], [61] and glucose metabolism [62], as well as using glucose metabolism or perfusion mapping in methyl-4-phenyl-1,2,3,6 tetrahydropyridine (MPTP) primates [63], [64]. Significant hyperactivation of the subthalamic nucleus was observed only in a small unilateral cluster at rest, different from the previously reported unilateral lesion model where prominent increases in rCBF were noted bilaterally [52]. Instead, our results showed a robust hyperactivity in the zona incerta (ZI) of lesioned rats, confirming earlier results of Perier et al. in the 6-OHDA rat [65].

In addition, lesioned compared to sham animals showed a functional reorganization in the cerebellum, including (3) increased rCBF in the vermis (rest), and deep cerebellar nuclei (during walking). Our results are consistent with human studies in PD showing that patients off medication may compensate for their basal ganglia-cortical loop’s dysfunction using motor pathways involving the cerebellum [66]–[69]. These findings suggest the possibility of a compensatory response, either via the cerebellar-thalamocortical circuit [70] or more directly as has been proposed via a dysynaptic pathway from the cerebellum to the striatum [71].

Lesioned compared to sham animals also showed (4) a significant functional recruitment of associated motor regions, including the superior colliculus and red nucleus (at rest and during walking), as previously reported by us in the unilateral lesion model [52]. The superior colliculus (part of the tecto-reticulospinal tract) plays a central role in the suppression of the eyeblink reflex, and its inhibitory modulation by the substantia nigra has been suggested to explain the increased eyeblink reflex reported in the 6-OHDA rodent model [72], [73], as well as in human PD subject [74]. In the red nucleus, a structure in the rostral midbrain involved in motor coordination, prior work has shown an increase in glucose utilization in striatally lesioned rats [75], as well as alterations in structure and function in PD subjects [76], [77].

Finally, lesioned rats demonstrated (5) a functional reorganization of the cortical map, with functional recruitment of broad regions of primary and secondary somatosensory cortex (at rest and during walking) and auditory cortex. Such a ‘diffusion’ or remapping of cortical sensorimotor activity has been previously reported in the unilateral 6-OHDA animal model [52], [78], [79] and suggests that somatosensory cortex may have to ‘work harder’ after striatal damage in order to provide adequate sensory processing to the animal, both at rest and during treadmill walking. Indeed others have reported somatosensory abnormalities in the 6-OHDA rat model [79], as well as somatosensory deficits in patients with PD [80], [81]. Consistent with this cortical remapping was an increase in the rCBF of the ventral posterolateral nucleus of the thalamus, a primary thalamic relay for somatic sensory, i.e. tactile/kinesthetic and nociceptive information from the trunk and limbs [82], which provides direct projections to the caudal striatum [83]. Increases were noted bilaterally, both at rest and during the locomotor challenge, and may indicate increased neural activity whose aim is to boost neural input to a hypofunctional striatum.

### Effects of Exercise Training (“Lesion/ET vs. Lesion/No-ET”) on Functional Activation of the Motorsensory System

Infusion of the 6-OHDA into the striatum causes immediate damage of local dopaminergic terminals, followed by retrograde loss over a period of 4–8 weeks of TH-positive cells in the substantia nigra compacta, resulting in persistent behavioral stereotypies [42], [84], [85]. This time course models aspects of the slowly evolving nature of the nigral lesion of PD and creates a therapeutic window for the investigation of potentially neurorestorative treatments, including exercise. Exercise training was initiated 2 weeks after 6-OHDA administration at a time when lesion maturation was well underway [42], [84], [85]. This allowed us to examine the neurorestorative effect of exercise, rather than its potential effects on attenuating acute neurotoxicity. Four weeks of daily, forced wheel running resulted in functional reorganization of the lesioned brain. During the locomotor challenge, exercised compared to nonexercised lesioned rats demonstrated significant increases in rCBF in the striatum (dorsal, anterior-medial), globus pallidus (internal, external), thalamus (reticular, ventral anterior, ventrolateral, ventromedial, ventroposterior lateral/medial), and pedunculopontine nucleus, as well as in rostral secondary motor cortex (“SMA”). However, rCBF in the midline cerebellum and deep cerebellar nuclei was decreased in exercised compared to nonexercised rats. Notably absent in the exercised lesioned animals in the current study was a significant bilateral decrease in relative perfusion in striatum and motor cortex clearly seen after ET in normal (nonlesioned) rats [86]. Such decreases have been previously suggested to reflect an increased bioenergetics capacity with a higher economy of neural processing in trained compared to untrained subjects [87]–[90]. This suggests that such efficiencies due to exercise may be attenuated following brain injury. The exercise-dependent increased activations of rostral secondary motor cortex and striatum were most clearly present in the walk-vs.-rest comparison (Fig. 7). Here nonexercised, lesioned animals showed a loss of the normal activation noted in sham rats. With ET, lesioned rats demonstrated a restoration of this activation. The walk-vs.-rest comparison also clearly showed a hyperemia of the zona incerta in nonexercised, lesioned rats–an effect that was absent after ET of the lesioned animals.

When we applied a seed analysis to the dorsal striatal region (data not shown) [91], exercise compared to no-exercise resulted in the appearance of new positive correlations with motor cortex (primary, secondary) and the ventrolateral thalamus. New negative correlations were noted in the deep nuclei of the cerebellum. The later finding underscores that exercise may elicit an overall increased recruitment of the cerebellar-thalamocortical circuit. Work in both animal [86], [92] and human subjects [93] indeed suggests that long-term ET results in a functional recruitment of the cerebellum.

### Functional Brain Mapping of the Motor Circuit: Rodent-to-Human Translation

Most human studies have examined activation of motor circuits in supine subjects during the performance of finger tasks, foot tasks or during imagining of movement. Evaluation of gait-induced brain circuitry activation has been performed in only a few studies using [^99m^Tc]-hexamethylpropyleneamine oxime SPECT or [^18^F]-fluorodeoxyglucose PET. Fukuyama et al. found in healthy subjects activation of the SMA, medial primary sensorimotor area, striatum, cerebellar vermis, and the visual cortex after walking compared to rest [15]. These findings closely match the activational pattern noted in our sham animals and underscore earlier observations that the basal ganglia have a highly conserved circuit architecture in vertebrates [94]. Hanakawa et al. performed a SPECT study during treadmill walking in PD patients and healthy control subjects. Active brain areas during walking in control subjects included the primary sensorimotor cortex, SMA, lateral premotor cortex, cingulate cortex and basal ganglia. Less activation was seen in the PD group in the right SMA, basal ganglia, left precuneus, and left cerebellar hemisphere, whereas they showed relative overactivity in the left temporal cortex, right insula, left cingulate cortex, and cerebellar vermis [95]. Our study showed similar changes in rCBF during treadmill walking in lesioned animals in the rostral M2, insula, and striatum, whereas the increase in rCBF in the vermis was noted only at rest.

As a rule of thumb, PD patients tend to show increased activity in motor cortical areas during the performance of overlearned motor tasks. Conversely, motor cortical areas tend to be underactive if the motor task demands a high amount of attentional guidance [96]. A cortical area consistently implicated in PD pathology is the SMA, although the findings are not entirely consistent. Several positron emission tomographic (PET) and functional magnetic resonance imaging (fMRI) studies have shown hypoactivation of the SMA in PD during movement, but others have shown the opposite (reviewed in [97]). Our study showed hypoactivation of the rostral M2 (rodent equivalent of the SMA) [57]–[59] in lesioned rats, suggesting a relatively high attentional demand for the horizontal treadmill task–a task to which rats had been familiarized, but which was not overlearned.

In recent years, PD has been associated also with alterations in disease-related networks [98], [99]. A PD-related metabolic pattern (PDRP) has been described using covariance analyses, and includes increased metabolic activity in the globus pallidus/putamen, thalamus, pons, and cerebellum, with associated relative reductions in the lateral premotor cortex, parieto-occipital association regions and SMA. This PDRP has been replicated in numerous studies comparing patients and healthy control subjects [98]. The pattern can be seen also using single photon emission computer tomography (SPECT) [100], [101], arterial spin labeling MRI [102] or [^15^O]-PET [103] to study cerebral blood flow. These findings parallel our finding in lesioned animals at rest (lesion vs. sham), which showed increased rCBF in the globus pallidus, ventrolateral thalamus, pons and cerebellum, with decreases in visual, parietal and rostral secondary motor cortex. Different were the decreases in rCBF noted directly at the dorsal striatal lesion site, though anterior and ventral aspects of the striatum did show bilateral increases in rCBF.

### Effects of Lesions and Exercise on Mesolimbic and Paralimbic Circuits

An extensive literature in human PD subjects documents the presence of mesolimbic dopaminergic deafferentation alongside alterations in mood, reward response, and amygdalar activation [24], [25]. In addition to processing motor information, an important interaction exist between the basal ganglia and the limbic system [23]. In our rodent model, dopaminergic deafferentation resulted in a significant increase in rCBF in a number of regions in the mesolimbic circuit, including broad areas of the amygdala, ventral hippocampus, ventral striatum, nucleus accumbens and tegmental nucleus (Fig. 5, tables 1–3). Also noted was a significant increase in rCBF in lesioned compared to sham-lesioned rats in the raphe, septum, insula, piriform and entorhinal cortex, and the dorsal endopiriform nucleus, with no significant difference noted in medial prefrontal cortex. Our results are consistent with the reported altered neural firing patterns and metabolism in the amygdala of the 6-OHDA rat [104], [105] and point to the involvement of the basal ganglia in limbic processing.

**Table 1:** Regions of statistically significant differences of functional cerebral cortical activation as determined by SPM analysis.

	Walk vs.Rest			Lesion vs.Sham		ET vs.No-ET	
	Lesion	Lesion	Sham	Walk	Rest	Walk	Rest
*CORTEX*	ET	No-ET	No-ET	No-ET	No-ET	Lesion	Lesion
**Auditory (Au)**	−/−	(−)/0	−/−	0/(+)	(+)/0	0/(−)	(+)/+
**Cingulate (Cg)**	+/+	+/+	+/+			−/−	(−)/0
**Dorsal peduncular cortex (DP)**	+/+	+/+	+/+	+/+	+/+	−/−	
**Ectorhinal (Ect)**	−/−		−/−	−/−	/−	(+)/(+)	(+)/+
**Entorhinal (Ent)**	+/+	+/+	+/+	+/+	+/+	+/(+)	+/+
**Infralimbic**	+/+	(+)/(+)	+/+			−/−	
**Insular (I)**	−/−	−/−	−/−	+/+	+/+	+/+	(−)/(−)
**Motor, primary (M1)**	+/+	+/+	+/+	−/−			−/(−)
** secondary (M2), rostral**	+/+	0/(+)	+/+	−/−	0/(−)	(+)/(+)	(−)/(−)
** secondary (M2), caudal**	+/+	+/+	+/+	−/−	(−)/−		(−)/(−)
**Orbital, lateral, ventral (LO, VO)**		+/+	(+)/(+)			−/−	
**Parietal assoc. Cx (PtA)**	+/+	+/+	+/+		0/−	(−)/(−)	−/0
**Piriform (Pir)**	−/−		(−)/(−)	+/+	+/+	(−)/(−)	(+)/(+)
**Prelimbic (PrL)**	+/+	(+)/(+)	+/0			−/−	(−)/(−)
**Retrosplenial (RS)**	+/+ a	+/+ a	+/+ a	0/−	−/−		0/+
		−/− p					
**Somatosensory, primary, dysgranular (S1DZ)**	+/+	+/+	+/+				−/(−)
** forelimb (S1FL)**			(+)/(+)	−/−	(+)/(+)	(−)/0	−/−
** trunk (S1Tr)**		−/−		−/(−)		−/−	0/(+)
** jaw (S1J)**	−/−	−/−	−/−	(+)/+	+/+	(+)/0	
** upper lip (S1ULp)**	−/−	−/−	−/−	0/(+)	+/+		
**Somatosensory, seconday (S2)**	−/− a,	−/− a,	−/− a,	+/(+) a	+/+ a, p		(−)/0 a
	+/+ p	+/+ p	+/+ p				
**Visual, primary (V1)**	(+)/(+)	(+)/(+)	+/+	−/(−)	+/0		
** secondary (V2)**	+/+	+/+	(+)/(+)	−/(−)	0/−		

Data are for rats with bilateral striatal lesions, sham lesions, during treadmill walking or at rest, and with or without a prior history of exercise training (Groups: Lesion/Walk/No-ET, n = 9; Sham/Walk/No-ET, n = 10; Lesion/Rest/No-ET, n = 10; Sham/Rest/No-ET, n = 9; Lesion/Walk/ET, n = 11; Lesion/Rest/ET, n = 12). Significance for all entries is shown at the voxel level (P<0.05) with a minimum extent threshold of 100 contiguous voxels. ‘a’ denotes ‘anterior’ and ‘p’ denotes ‘posterior’. ‘+’ denotes increases and ‘−’ denotes decreases in the group differences in cerebral blood flow tracer distribution in the left/right hemispheres, with symbols in parentheses denoting changes less broadly represented.

**Table 2:** Regions of statistically significant differences of functional subcortical activation as determined by SPM analysis.

	Walk vs.Rest			Lesion vs.Sham		ET vs.No-ET	
	Lesion	Lesion	Sham	Walk	Rest	Walk	Rest
*SUBCORTEX- MOTOR RELATED*	ET	No-ET	No-ET	No-ET	No-ET	Lesion	Lesion
**Cerebellum, vermis (1st lobule, 1Cb)**	+	+	+	–		–	–
** vermis (2^nd^, 3^rd^, 4^th^ lobules, 2Cb, 3Cb**, **4Cb)**	+	+	+	−3Cb	+	–	–
**Cerebellar deep n. (medial, lateral, interposed)**	(−)/(−)	(+)/(+)		+/+	+/(+)	−/−	
**Colliculus, superior (SC)**	−/−	−/−	−/−	+/+	+/+		−/−
**Globus pallidus, external/lateral (GPe)**	(−)/(−)	−/−	(−)/(−)	(+)/(+)	+/+	+/+	(−)/(−)
**Globus pallidus internus, entopeduncular n. (GPi)**	(−)/(−)	−/−	(−)/(−)	(+)/0	+/+	+/+	
**Pedunculopontine tegmental n. (PTg)**		−/−	−/−			+/+	
**Red n., pararubral n. (RPC, PaR)**	(−)/(−)	(−)/−	−/(−)	+/+	+/+		−/(−)
**Striatum, anterior-medial (ant**-**CPu)**	(+)/(+)	−/−	+/+	−/−	+/+	+/+	+/+
** dorsal (d**-**CPu)**	+/+	(−)/0	+/+	−/−	−/−	+/+	−/(−)
**Substantia nigra (SN)**	−/−	−/−	−/−		(+)/0		
**Subthalamic n. (STN)**	(−)/(−)	(−)/(−)	(−)/(−)		0/(+)		
**Trigeminal n., motor, sensory (5)**	−/−	−/−	−/(−)	+/(+)	(+)/+	(+)/0	
**Zona incerta (ZI)**		+/+		0/(+)	(+)/(+)	0/(−)	(+)/(+)
***SUBCORTEX- THALAMUS***							
**Thalamus, anterior medial (AM)**	−/−	(−)/(−)		−/−			
** central medial (CM)**	–	–	–	+	+		
** habenula (Hb)**			(+)/+		+/+	−/−	−/−
** posterior n. (Po)**			+/+		+/+		
** reticular (Rt)**	−/−	−/−		−/−		+/+	−/(−)
** ventral anterior n. (VA)**		−/−			+/+	(+)/+	
** ventrolat. n. (VL)**	−/−	−/−	−/−	(−)/0	+/+	(+)/+	
** ventromed. n. (VM)**	−/−	−/−	−/−	+/+	+/+		
** ventropost. lat., ventropost. med. (VPL, VPM)**		−/−		+/+	+/+	+/+	
***SUBCORTEX- LIMBIC & LIMBIC RELATED***							
**Amygdala, central n. (Ce)**	−/−	−/−	−/−	/(+)	+/+	+/+	−/(−)
** basolateral n. (BL)**	−/−	−/−	(−)/(−)	/(+)	+/+	+/+	
** lateral n. (La)**	−/−	−/−	(−)/(−)	−/−		+/+	0/(+)
** medial n. (Me)**	(−)/(−)		−/−	+/+	0/(−)	(−)/−	−/(−)
** cortical (Aco, PMCo)**		+/+	−/−	+/+	(−)/(−)	(−)/−	(+)/(+)
**Amygdaloid**-**piriform trans. (APir), amygdaloid**-** hippoc. trans. (AHi)**		+/+	−/−	+/+	+/(+)	(−)/−	(+)/(+)
**Endopiriform n., dorsal (DEn)**			−/−	+/+	0/(−)	(−)/(−)	(+)/+
**Hippocampus, dorsal (d-HPC)**	+/+	+/+	+/+	−/−	−/−	+/+	(−)/(−)
**Hippocampus, ventral (v-HPC)**	+/+	+/+	+/+	+/+	(+)/0		+/+
**Hypothalamus, ventromed. (VMH)**	(−)/(−)		−/−		−/−		
**Nucleus Accumbens (Acb)**	(−)/−		−/−	(+)/(+)	(+)/(+)		
**Periaqueductal gray, lateral (LPAG)**	−/−	−/−	−/−			0/(+)	(+)/(+)
**Raphe, median, paramedian (MnR, PMnR)**	–	(−)		+	+	(−)	
**Septum, medial (MS)**	(+)	+	+	+	+	–	(−)
** lateral (LS)**	(+)/(+)	+/+	(+)/(+)	+/+	+/+	−/−	(+)/(+)
**Striatum, ventromedial/ventrolateral (v**-**CPu)**	−/−	−/−	−/−	(+)/0	+/+	(+)/(+)	
**Subiculum, dorsal (DS)**	+/+	+/+	+/+				
** post (Post)**	(+)/(+)	−/(−)	+/+	−/−	(+)/(+)	+/+	
** ventral (VS)**	+/+	+/+	+/+	0/(+)		(−)/−	+/+
**Tegmental n., dorsal, laterodorsal (DTg, LDTg)**				+/+	+/+	(−)/(−)	(−)/(−)
**Ventral pallidum (VP)**	−/−		−/−	(+)/(+)		(+)/(+)	+/+
***SUBCORTEX- OTHER***							
**Colliculus, inferior (IC)**	−/−	−/−		(−)/0	+/+	(+)/(+)	0/+
**Cuneiform n. (Cn)**	0/−		−/−	+/(+)			
**Gigantocellular n. (GPi)**	(−)/(−)	(−)/(−)	(−)/(−)	+/+	+/+		
**Interpeduncular n. (IP)**	–	–	–	+	+	(−)	(−)
**Parvocellular reticular n. (PCRt)**	−/−		−/−	+/+			
**Pons (Pn)**	(−)/(−)	−/(−)	(−)/(−)	(+)/(+)	(+)/(+)		(−)/0
**Reticular formation, mesencephalic (mRt, Cn)**	−/−	−/−	−/−			(+)/0	(+)/(+)
**Tenia tecta, navicular n. (TT/Nv)**			−/−	+/+	+/+	−/−	
**Vestibular n. (Ve)**	−/−			+/+	+/+	−/−	(+)/(+)

Data are for rats with bilateral striatal lesions, sham lesions, during treadmill walking or at rest, and with or without a prior history of exercise training (Groups: Lesion/Walk/No-ET, n = 9; Sham/Walk/No-ET, n = 10; Lesion/Rest/No-ET, n = 10; Sham/Rest/No-ET, n = 9; Lesion/Walk/ET, n = 11; Lesion/Rest/ET, n = 12). Significance for all entries is shown at the voxel level (P<0.05) with a minimum extent threshold of 100 contiguous voxels. ‘a’ denotes ‘anterior’ and ‘p’ denotes ‘posterior’. ‘+’ denotes increases and ‘−’ denotes decreases in the group differences in cerebral blood flow tracer distribution in the left/right hemispheres, with symbols in parentheses denoting changes less broadly represented.

**Table 3:** Factorial analysis showing regions of statistical significant differences in functional brain activation.

	Lesion	Lesion×Walk	Exercise	Exercise×Walk
**Basal ganglia thalamocortical** **circuit**	CPu (anterior, dorsal), GPe,GPi, M2 (rostral, caudal)	CPu (anterior, dorsal), M1,M2 (rostral), VL	CPu (dorsal), GPi, M2(rostral), VL	CPu (anterior, dorsal), GPe,GPi, M1, M2 (rostral), VL
**Associated motor areas**	RPC, SC, trigeminal n.(motor/sensory), ZI	SC	PTg, SC	PTg, RPC, SC, ZI
**Cerebellar-thalamocortical** **circuit**	deep cerebellar nuclei(medial, lateral, interposed),vermis	vermis, VL	vermis, VL	deep cerebellar nuclei (medial,lateral, interposed), VL
**Somatosensory**	Ect, S1FL, S1J, S1UL, S2	Ect, S1FL, S1Tr, S2, V1	Ect, PtA, S1FL	Au, PtA, S1DZ, S1Tr
**Motor-sensory** **thalamic nuclei**	CM, VL, VM, VPL/VPM	VA	Hb, VA, VL	VA, VL, VPL/VPM, Rt
**Mesolimbic/paralimbic**	Acb, Amygdala (BL, Ce, APir,AHi), DEn, dHPC, Ent, insula,Pir, raphe, RS, septum(MS, LS), tegmental n. (DTg, LDTg),vCPu, vHPC	Acb, Amygdala (BL, Ce, LA,Me, cortical, APir, AHi), DEn,dHPC, insula, Pir, subiculum (Post,VS), RS, vCPu, VMH,vHPC	Acb, Amygdala (BL, LA),dHPC, Ent, mPFC (Cg, PrL, IL),MS, Pir, RS, subiculum (Post, VS),tegmental n. (DTg, LDTg),vCPu, VP	Amygdala (BL, Ce, Me,APir, AHi), DEn, dHPC, DEn,Ent, insula, LPAG, LS, orbitalcx (LO, VO), subiculum (Post, VS),vHPC

Shown is the main effect in non-exercised rats of Lesion and the Lesion×Walk interaction (Groups: Lesion/Walk/No-ET, n = 9; Sham/Walk/No-ET, n = 10; Lesion/Rest/No-ET, n = 10; Sham/Rest/No-ET, n = 9). Also shown is the main effect in lesioned rats of Exercise and the Exercise×Walk interaction (Lesion/Walk/No-ET, n = 9; Lesion/Rest/No-ET, n = 10; Lesion/Walk/ET, n = 11; Lesion/Rest/ET, n = 12). Significance (*) for all entries is shown at the voxel level (P<0.05) with a minimum extent threshold of 100 contiguous voxels. Abbreviations are those from the Paxinos and Watson rat atlas [39]: 5 (trigeminal n., motor, sensory), aca (anterior commissures), AHi (amygdalo-hippocampal area); APir (amygdalo-piriform transition area); BL (basolateral amygdala), Ce (central amygdalar n.), Cg (cingulate cortex), CM (central medial thalamic n.), CPu (striatum: anterior, ant-CPu; dorsal, d-CPu; ventral, v-CPu), d-HPC (dorsal hippocampus), DTg/LDTg (dorsal/laterodorsal tegmental n.), Ent (entorhinal cortex), GPe (external globus pallidus), GPi (internal globus pallidus/entopeduncular n.), Hb (habenula), I (insular cortex), IL (infralimbic cortex), La (lateral amygdala), LO (lateral orbital cortex), LP (lateral posterior thalamic n.), LS (lateral septum), M1, M2 (primary, secondary motor cortex), Me (medial amygdala), MS (medial septum), Pir (piriform cortex), PrL (prelimbic cortex), PtA (parietal association cortex), PTg (pedunculopontine tegmental n.), RPC (red n.), RS (retrosplenial cortex), S1DZ, S1FL, S1J, S1Tr, S1ULp, (primary somatosensory cortex: dysgranular, forelimb, jaw, trunk, lip), S2 (secondary somatosensory cortex), SC (superior colliculus), vermis (2^nd^, 3^rd^ cerebellar simple lobule), V1, V2 (primary, secondary visual cortex), v-HPC (ventral hippocampus), VL (ventral lateral thalamic n.), VM (ventromedial thalamic n.), VMH (ventromedial hypothalamus), VPL/VPM (ventral posterolateral, ventral posteromedial thalamic nuclei), VP (ventral pallidum), VS (ventral subiculum), ZI (zona inserta).

**Figure 6:**
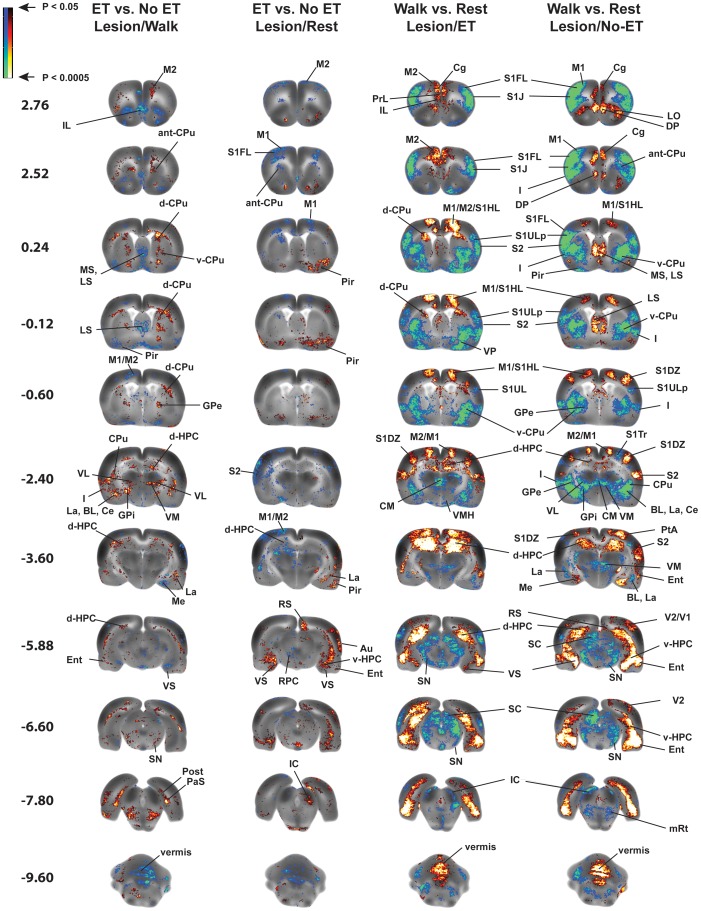
Effects of prior exercise training (ET) on functional brain activation. Shown are statistically significant differences in activation during acute treadmill walking (ET/Walk, *n* = 11, No-ET/Walk, *n* = 9) or at rest (ET/Rest, *n* = 12, No-ET/Rest, *n* = 10) in rats with bilateral striatal lesions. Depicted is a selection of representative coronal slices (anterior–posterior coordinates relative to bregma). Colored overlays show statistically significant positive (red) and negative (blue) differences (voxel level, *P*<0.05). Abbreviations are those noted in the legend of Fig. 5.

While a growing literature has documented beneficial effects of exercise on depressive and anxiety symptoms in both humans and animals [106]–[108], few studies have examined effects of long-term exercise on activation of the limbic system outside the hippocampus [90], [109]. In our study, ET in lesioned animals, compared to no-ET elicited functional changes in the mesolimbic system (Fig. 6, tables 1–3). These included significant increases in rCBF during treadmill walking in the amygdala (central, basolateral, lateral), dorsal hippocampus, ventral/ventrolateral striatum, and insula, and decreases in rCBF in the amygdala (medial, cortical, amygdaloid-hippocampal and amygdaloid-piriform transition area), as well as septum (medial, lateral) and piriform cortex. When imaged at rest, however, exercise effects were reversed in these regions, emphasizing that regional activity patterns are highly task dependent and likely modulated by attentional processes. As recently suggested by Greenwood et al [110], our results support the notion that regions such as basolateral amygdala and striatum may be novel targets for the anxiolytic and antidepressant effects of exercise.

In addition, ET resulted in broad, significant decreases in rCBF in the midline frontal/prefrontal cortex, including cingulate, prelimbic and infralimbic cortex. This finding is consistent with our prior results in nonlesioned rats [86], as well as work in human imaging showing decreased glucose uptake in frontal and cingulate cortex during motor exertion in subjects with a prior history of ET compared to those with limited training [111]. Of note, prelimbic cortex in the rodent shows partial homology with the dorsolateral prefrontal cortex of primates [112], [113], a brain region that shows increased activation after acute exercise [114] but may reduce its activity after prolonged physical training, a finding that has been speculated to reflect increased brain efficiency [115].

## Conclusion

Current research in PD is dominated by a focus on the basal ganglia and an emphasis that symptoms are determined by cell death. While the importance of these concepts cannot be understated, their elevation to the status of dogma has hindered a broader understanding of the illness and its treatment. Our results highlight the importance of functional reorganization following dopaminergic deafferentation that involve regions within the basal ganglia-thalamocortical, cerebellar-thalamocortical, as well as in associated motor, sensory and thalamic structures. It is likely that such changes reflect a combination of cell death, cell dysfunction, as well as compensatory functional recruitment. Though our study does not allow differentiation between these factors, it begins to lay the groundwork for understanding the observation that animals following brain injury learn to engage in alternative behavioral strategies that lead to a greater dependence on less compromised motor systems [116]–[119] – an observation also noted in human subjects [120], [121]. Future studies may wish to evaluate how experience-specific reorganization of the brain may aid or impede the acquisition of new behaviors or the performance of old behaviors. Such proactive and retroactive interference of motor behaviors has long been recognized but are lacking a sound scientific explanation [1], [119], [122], [123].

Few studies have examined the effects of long-term aerobic training on functional brain activation in PD subjects [21], [22]. Our study uniquely showed that 4 weeks of ET elicited a functional reorganization in the basal ganglia-thalamocortical circuit, including increased activation of the dorsal striatum and rostral secondary motor cortex, as well as attenuation of the hyperemia of the zona incerta noted in nonexercised, lesioned animals. In addition, exercise elicited a functional reorganization of regions participating in the cerebellar-thalamocortical circuit. Finally, both lesions and exercise broadly increased activation with the mesolimbic circuit, as well as related paralimbic regions. The exercise-dependent functional reorganization of the mesolimbic circuit may be of relevance to those proposing exercise as a therapeutic intervention for patients suffering from mood disorders, including PD subjects [106], [124]. Given the close parallels between the 6-OHDA lesion model and PD [26] and the fact that ET was initiated during a period of progressive retrograde cell loss in the substantia nigra compacta [42], [84], [85], we suggest that the exercise-dependent functional reorganization of the brain in our study models what one might expect to see in a chronic progressive neurodegeneration such as PD.
